# Change in knee structure and change in tibiofemoral joint space width: a five year longitudinal population–based study

**DOI:** 10.1186/s12891-016-0879-0

**Published:** 2016-01-14

**Authors:** Joanna Hall, Laura L. Laslett, Johanne Martel-Pelletier, Jean-Pierre Pelletier, François Abram, Chang-Hai Ding, Flavia M. Cicuttini, Graeme Jones

**Affiliations:** Menzies Institute for Medical Research, University of Tasmania, Private Bag 23, Hobart, Tasmania 7000 Australia; Osteoarthritis Research Unit, University of Montreal Hospital Research Centre (CRCHUM), Montreal, Quebec Canada; Imaging Research and Development, ArthroLab Inc., Montreal, Quebec Canada; Department of Epidemiology and Preventive Medicine, School of Public Health and Preventative Medicine, Monash University, Alfred Hospital, Prahran, 3181 Melbourne Australia; Arthritis Research Institute, 1st Affiliated Hospital, Anhui Medical University, Hefei, Anhui China

**Keywords:** Cartilage, Meniscus, Extrusion, X-ray, MRI

## Abstract

**Background:**

Change in knee cartilage volume is frequently used as a proxy for change in knee joint space width over time, but longitudinal data on these associations is limited. We aimed to determine whether change in knee cartilage volume, new or worsening meniscal extrusion (ME), meniscal tears and cartilage defects over 2.4 years correlated with change in joint space width (JSW) over 5 years in older community dwelling adults.

**Methods:**

Participants (*n* = 153) had their right knee imaged using MR imaging and x-ray at baseline, and after 2.4 years (MRI) and 5 years (x-ray). Cartilage volume, cartilage defects, meniscal extrusions and meniscal tears were assessed on sagittal T1-weighted fat-suppressed MRI. JSW was assessed using standard fixed semi-flexed view radiographs, and scored on those with adequate alignment.

**Results:**

Participants were 51–79 (mean 62) years old; 48 % were female. Cartilage volume reduced over time (medial −134 ± 202 μL/year, lateral −106 ± 165 μL/year, *p* < 0.001), as did JSW (medial −0.05 ± 0.16 mm/year, lateral −0.12 ± 0.24 mm/year, *p* < 0.001). In multivariable analysis, the only consistent predictor of change in JSW was new or worsening ME (medial tibia R^2^ 3.1 %, *p* = 0.031; medial femur R^2^ 3.2 %, *p* = 0.024); change in cartilage volume correlated with change in JSW laterally (R^2^ 4.8 %, *p* = 0.007) and was borderline medially (R^2^ 2.2 %, *p* = 0.064); there was no association for meniscal tears or cartilage defects. The magnitude of these associations were similar albeit somewhat greater for ME in participants with radiographic OA (R^2^ 6.2 %, *p* = 0.017).

**Conclusion:**

Change in ME and cartilage volume weakly predict change in JSW, but the vast majority of the variation remains unexplained. Since MRI examines cartilage directly while radiographs examine it indirectly, these results cast doubt on the validity of using JSW as a proxy measure of cartilage loss.

## Background

Osteoarthritis (OA) is a major cause of pain and functional limitations and disability worldwide [1]. Diagnosis is based on a combination of symptoms, clinical signs and radiographic abnormalities [2]. Change in joint space width (JSW) at the tibiofemoral joint has historically been considered a good measure of change in cartilage volume. It is currently the gold standard for assessing osteoarthritis disease modification in clinical trials [3], and is mandated by the Food and Drug Administration and European Medications Agency as a proxy endpoint to determine efficacy of disease modifying osteoarthritis drugs. In cross-sectional studies, cartilage volume assessed by magnetic resonance imaging (MRI) and JSW as assessed by radiograph are strongly correlated. However, JSW is also associated with meniscal pathology [4–7], and cartilage defects [8], suggesting that multiple abnormalities contribute to narrowing of joint space width (JSW) over time. The presence of radiographic OA also predicts patients who lose cartilage faster; this has implications for participant selection in clinical trials [9, 10].

A key remaining question is whether radiographic JSW is the most appropriate measure for assessing change in knee OA over time. Some studies have shown significant structural change at the tibiofemoral joint over time, such as trials involving glucosamine, doxycycline and chondroitin [11–14]. However, in a trial investigating the effect of risedronate on OA progression, there was minimal change in the placebo arm over 2 years, despite large numbers of patients and state of the art protocols [15]. MRI-based cartilage loss has greater sensitivity to change [16–18]; than change in radiographic JSW [19]. However, these assessments come from different cohorts; there is limited longitudinal data comparing change in radiographs with change in MRI within cohorts. Two studies reported weak but statistically significant correlation between changes in JSW and cartilage volume at 1 year in OA cohorts, using the fixed-flexion radiographic techniques [17, 20]. Both Cicuttini and Raynauld reported no correlation between x-ray change and cartilage volume change on MRI over 2 years, using different methodologies: standing protocol radiographs [21] and a fluoroscopically guided AP semiflexed protocol [22]. Hunter et al [23] assessed predictive value of cartilage score on change in medial JSN in patients with symptomatic knee OA over 30 months, using fluoroscopic positioning and fixed flexion. They found that cartilage score contributed to change in JSN beyond age sex and BMI, but that most of the variability in JSN remained unexplained [23]. Since MRI has been directly validated for cartilage volume measurement, this raises the possibility that JSW is not a sufficiently adequate measure of cartilage to qualify it for use as an outcome measure in clinical trials.

Moreover, data in the literature also reported that meniscal position and cartilage morphology score also contribute to variance in JSW [7, 23]. However, to our knowledge there have been no prospective studies which have determined the quantitative contribution of meniscal extrusion to narrowing of JSW over a period as long as 5 years. Therefore, this study aimed to determine whether change in cartilage volume and other structural factors, including new or worsening meniscal extrusion (ME), worsening meniscal tears, and worsening cartilage defects over 2.4 years predicted change in JSW over 5 years in participants from a randomly selected cohort of community dwelling older adults with and without radiographic OA.

## Methods

### Study design and setting

The Tasmanian Older Adult Cohort (TASOAC) study is an ongoing prospective population–based study in southern Tasmania, Australia, which began in 2002.

### Study participants

Men and women aged 50–80 years were randomly selected from the roll of electors in southern Tasmania (population 229,000), a comprehensive population listing, using sex-stratified simple random sampling without replacement (response rate 57 %). Permission to access the roll of electors was granted by the Australian Electoral Commission. Persons were excluded if they were institutionalized, or had contraindications to MRI. The study was approved by the Southern Tasmanian Health and Medical Human Research Ethics Committee, and written informed consent was obtained from all participants. Baseline measurements (Phase 1) were conducted from April 2002 to September 2004. Follow up data (Phase 2 and 3) was collected 2.4 (range 1.7 to 2.9) and 5 years (range 4.6 to 5.9) later.

The current study consists of a sample of 153 participants who had data for x-rays and MRI at baseline and follow-up with adequately aligned radiographs (see Table 1).

**Table 1:** Participant characteristics

		Mean (SD) *n* = 153
Gender		48 % female
Age (years)		62.2 (7.0)
BMI (kg/m^2^)		27.8 (4.7)
Any radiographic OA (%)		56.0
Osteophyte, any site (%)		9.2
Medial JSW (mm)		4.73 (0.97)
Lateral JSW (mm)		6.76 (1.37)
JSN scores (OARSI grade) (%)		
Medial	0	49.7
	1	41.2
	2	8.5
	3	0.7
Lateral	0	83.7
	1	13.1
	2	2.0
	3	1.3
Annual change in joint space width:		
Medial (mm)		−0.05 (0.16)
Medial (%)		−1.2 (3.9)
Lateral (mm)		−0.12 (0.24)
Lateral (%)		−1.8 (3.9)
Medial cartilage volume (tibia + femur, μL)		6296 (1523)
Lateral cartilage volume (tibia + femur, μL)		7080 (1671)
Annual change in cartilage volume:		
Medial (μL)		−134 (202)
Medial (%)		−2.1 (3.0)
Lateral (μL)		−106 (165)
Lateral (%)		−1.6 (2.5)
Meniscal extrusions		
Medial – present at baseline (%)		16.3
Medial – worsening extrusion (%)		9.2
Lateral – present at baseline (%)		2.0
Lateral – worsening extrusion (%)		0.7
Meniscal tears		
Medial – present at baseline (%)		98.0
Medial – worsening tear (%)		10.5
Lateral – present at baseline (%)		96.7
Lateral – worsening tear (%)		15.0
Cartilage defects		
Medial - present at baseline (%)		23.5
Medial - worsening (%)		37.7
Lateral - present at baseline (%)		21.6
Lateral - worsening (%)		36.4
Cartilage thickness (average, tibia & femur)		(*n* = 67)
Medial (mm)		3.07 (0.41)
Lateral (mm)		3.33 (0.41)
Annual change in cartilage thickness		
Medial (mm)		−0.17 (0.27)
Medial (%)		−6.04 (8.45)
Lateral (mm)		−0.26 (0.3)
Lateral (%)		−8.12 (8.69)

Change in cartilage and meniscal extrusion is over a time period of 2.4 years

Change in joint space width is over a time period of 5 years

Right knees only

### Outcome measures

#### Knee radiographs

Standing anteroposterior semiflexed right knee radiographs with 10–15° of fixed knee flexion were performed at baseline and after 5 years. Radiographs were viewed on Osiris software (University Hospital of Geneva, Switzerland) and scored paired by one investigator (JH, after instruction from GJ), blinded to MRI data but not to chronological order. Measurements of the minimum JSW (mJSW) at the lateral and medial compartment were performed to the nearest 0.1 mm, using 200 % magnification and a digital calliper in the image processing package, and enhancement to improve cortical demarcation. mJSW was determined as the narrowest, non-osteophytic space of the lateral and medial compartments of the right knee using a modified version of the method of Lequesne [24], and using bony margins as described by Buckland-Wright. [25] Briefly, the femoral boundaries were the distal convex margin of the femoral condyles, and the tibial boundaries the bright radiodense band of the subchondral cortex of the tibia. The reader selected a minimum of five points in each compartment, with the smallest reading used. This technique is similar to the “calculated minimum” technique, which is more accurate than measurement of the narrowest site using visual assessment alone [26]. Intraclass correlation (ICC) was excellent, ranging from 0.92–0.99 in a random sample of 20 participants separated in time.

In the absence of fluoroscopic guidance, radiographs were limited to those which were well aligned (67 % of those with films at both time points) [27], defined as tibial inter-rim distance of the medial tibial plateau varying by ≤2 mm between films, measured at the midpoint of the medial compartment.

X-rays were scored individually for osteophytes and narrowing of JSW on a scale of 0–3 (0 = normal, 3 = severe) according to the Osteoarthritis Research Society International (OARSI) atlas [28] as previously described [29]. Intraobserver repeatability was acceptable (ICCs of 0.65 to 0.85 in 40 participants) [29]. The presence of radiographic OA was defined as any score of ≥1.

#### Magnetic resonance imaging

Magnetic resonance imaging (MRI) of the right knee was acquired using a 1.5 Tesla whole-body MRI unit (Picker, Cleveland, OH, USA) using a commercial transmit-receive extremity coil.

**Fig. 1:**
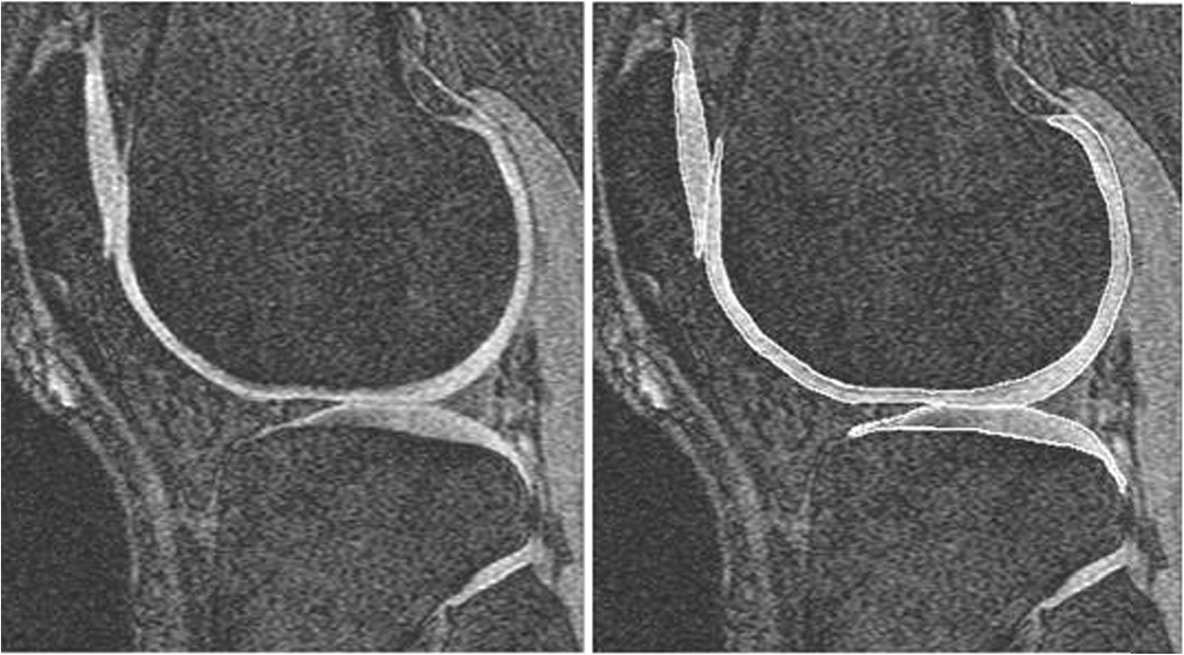
A representative MR image of cartilage segmentation. The volumes of individual cartilage plates (medial tibia and lateral tibia) were isolated from the total volume by manually drawing disarticulation contours around the cartilage boundaries on a section by section basis, before being re-sampled for 3-D rendering

Tibial cartilage volume was assessed at baseline and follow up by a trained observer on Osiris software as previously described [30, 31] using T1-weighted fat suppressed 3-dimensional gradient recall acquisition in the steady state, flip angle 55°, repetition time 58 msec, echo time 12 msec, field of view 16 cm, 60 partitions, 512 × 512–pixel matrix, acquisition time 11 min 56 s, and 1 acquisition. Sagittal images were obtained at a partition thickness of 1.5 mm and in-plane resolution of 0.31 × 0.31 mm (512 × 512 pixels). The volumes of individual cartilage plates (medial tibia and lateral tibia) were isolated from the total volume by manually drawing disarticulation contours around the cartilage boundaries on a section by section basis (see Fig. 1 for an image of representative segmentation). These data were then re-sampled by means of bilinear and cubic interpolation (area of 312 × 312 mm and 1.5 mm thickness, continuous sections) for the final 3-D rendering. The coefficient of variation (CV) was 2.1 % for the medial tibia and 2.2 % for the lateral tibia [30].

Femoral cartilage volume was determined by means of image processing on an independent workstation using Cartiscope™ (ArthroVision Inc., Montreal, Quebec, Canada), as previously described [4, 32, 33]. The segmentation of the cartilage-synovial interfaces was carried out using a semi-automatic method under reader supervision, and with corrections when needed. Cartilage volume was evaluated directly from a standardized view of 3D cartilage geometry as the sum of elementary volumes. The CV was approximately 2 % [32]. The cartilage volume assessment was done for the medial and lateral condyles delineated by the Blumensaat’s line [33].

Meniscal extrusion and tears were assessed using the T1-weighted fat suppressed sequences described earlier [4]. Anterior and posterior horns of the menisci were scored using the sagittal views and the body of the menisci using reconstructed coronal views. The proportion of the menisci affected by extrusion was measured separately on the medial and lateral edges of the tibiofemoral joint space using a semi-quantitative scale. The extent of meniscal extrusion, excluding osteophytes, was evaluated for the anterior, middle, and posterior horns of the menisci in which 0 = no extrusion, 1 = partial extrusion and 2 = complete extrusion with no contact with the joint space (severe) [4]. The extent of meniscal tears were assessed using the following scale: 0 = no damage; 1 = one of three meniscal areas involved (anterior, middle, and posterior horns); 2 = two of three areas involved; 3 = all three areas involved [4]. Change in meniscal tears were classified as improvement or no change if scores were unchanged or improved at either tibia or femur, or classified as worsening if scores increased. Reliability was excellent; intra- and inter-observer correlation coefficients ranged from 0.86–0.96 [22].

Mean cartilage thickness for each of four regions (femur facing the medial tibia, femur facing the lateral tibia, medial tibia and lateral tibia) was assessed using custom semi-automated segmentation software, and was calculated as the mean distance from inner to outer surface. This was done from a sample of uniformly spaced points over the entire cartilage-covered surface. Reliability was excellent: intra–observer reproducibility for mean intensity in each region was less than 1.5 %, with a coefficient of variation <2.9 % [34].

Cartilage defects were assessed by a trained observer at the medial tibial, medial femoral, lateral tibial, and lateral femoral sites, using the T1-weighted fat suppressed sequences described earlier, and as previously described [8]: grade 0 = normal cartilage; grade 1 = focal blistering and intracartilaginous low-signal intensity area with an intact surface and base; grade 2 = irregularities on the surface or base and loss of thickness <50 %; grade 3 = deep ulceration with loss of thickness >50 %; and grade 4 = full-thickness chondral wear with exposure of subchondral bone. ICCs ranged from 0.89–0.94 for intra-observer reliability. These were dichotomised into none (grades 0 and 1 (normal/focal blistering)) or any defects (grades 2 and above). Change in cartilage defects were classified as improvement or no change if scores were unchanged or improved on the 0–4 scale at either tibia or femur, or classified as worsening if scores increased.

### Statistical analysis

We used Stata 12.1 (Stata Corp LP) for statistical analyses. Statistical significance was set as a *p* value of ≤0.05 (two tailed). Linear regression was used to assess the association between change in cartilage volume, meniscal extrusions, meniscal tears over 2.4 years and change in JSW over 5 years. R^2^ values for univariable models are the R^2^ statistic from the linear regression. R^2^ values for multivariable models are the squared semipartial correlations for each individual predictor in the multivariable model, which represent the proportion of variance in the outcome that is explained by the individual predictor only. Total R^2^ is the sum of the squared semipartial correlations for individual predictors, and is given by the adjusted R^2^ statistic (adjusted for degrees of freedom) in the multivariable linear regression.

## Results

**Fig. 2:**
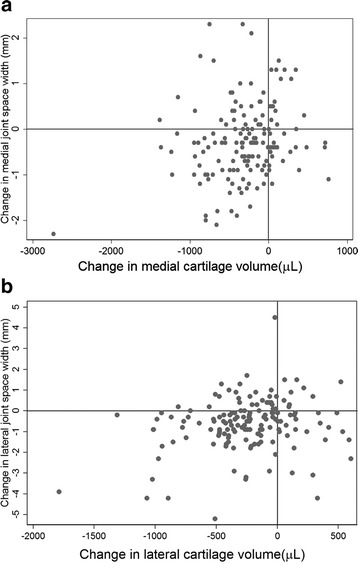
Scatterplots showing the association between change in joint space width (JSW) and change in cartilage volume (*n* = 153 medial, *n* = 152 lateral). In both compartments, cartilage volume decreased in 117 participants each of a and b, but in 29 in a and 26 in b JSW increased despite a decrease in cartilage volume. **a** Medial compartment: there is a significant negative correlation, with R^2^ = 4.4 %, *p* = 0.009 (unadjusted). **b** Lateral compartment: there is a significant negative correlation, with R^2^ = 4.9 %, *p* = 0.006 (unadjusted)

A total of 153 participants (48 % female, mean age 62 years, [range 51–79]) had adequately aligned radiographs (206 /307 pairs), complete MR imaging, and radiograph data of the knee at baseline and follow up. Baseline characteristics of this sample (*n* = 153) were similar to the overall TASOAC population (*n* = 1099) (age 62.2 vs 63.2 years, *p* = 0.14; sex 48 % female vs 49 % female, *p* = 0.75; BMI 27.8 vs 27.9, *p* = 0.84) (Table 1). Half of the participants (56 %, *n* = 84) had at least grade 1 radiographic OA at baseline, defined as either JSN or presence of osteophytes using the OARSI atlas [28]. Medial and lateral cartilage volume significantly reduced over time (medial −134.3 μL/year (95 % CI −166.6 to −102.0), lateral −106.2 μL/year, (95 % CI −132.5 to −79.9), as was JSW (medial −0.05 mm/year (95 % CI −0.08 to −0.03), lateral −0.12 mm/year, (95 % CI −0.16 to −0.08). Although the majority of participants demonstrated a decrease in cartilage volume over time, JSW both decreased and (Fig. 2). In the medial compartment, 67 % of participants had a decrease in JSW, 5 % stayed the same, and 28 % increased. Similar values were found for the lateral compartment (73 % had a decrease in JSW, 2.6 % stayed the same, and 24 % increased).

**Fig. 3:**
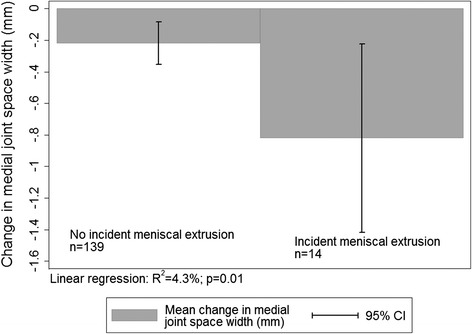
Mean change in joint space width stratified by meniscal extrusion. In participants with incident meniscal extrusion at 2.4 years there was a significantly greater decrease in JSW over 5 years (unadjusted)

Change in cartilage volume over 2.4 years was a significant predictor of change in JSW over 5 years in univariate analyses (R^2^ = 4.4 %, *p* = 0.009 medially; R^2^ = 4.9 %, *p* = 0.006 laterally) (Table 2, Fig. 2a and b). However, in multivariable analysis, change in cartilage volume was a significant predictor of change in JSW only in the lateral compartment (R^2^ 4.8 %, *p* = 0.007 laterally; R^2^ 2.2 %, *p* = 0.064 medially) (Table 2).

**Table 2:** Association between change in cartilage volume, and new or worsening meniscal extrusions, meniscal tears, and cartilage defects over 2.4 years; and change in joint space width over 5 years

Population	Change in..	Medial joint space width		Lateral joint space width	
		Univariable	Multivariable^a^	Univariable	Multivariable^a^
All participants	Tibial + femoral cartilage volume	** R** ^**2**^ **4.4 %,** ***p*** **= 0.009**	R^2^ 2.2 %, *p* = 0.064	** R** ^**2**^ **4.9 %,** ***p*** **= 0.006**	** R** ^**2**^ **4.8 %,** ***p*** **= 0.007**
(*n* = 151 medially,	Meniscal extrusion	**R** ^**2**^ **4.3 %,** ***p*** **= 0.01**	R^2^ 2.4 %, *p* = 0.053	NA	NA
*n* = 150 laterally)	Meniscal tears	R^2^ 0.19 %, *p* = 0.60	R^2^ 0.0 %, *p* = 0.97	R^2^ 1.4 %, *p* = 0.15	R^2^ 1.2 %, *p* = 0.18
	Cartilage defects (tibia + femur)	R^2^ 1.4 %, *p* = 0.15	R^2^ 0.7 %, *p* = 0.29	R^2^ 0.26 %, *p* = 0.54	R^2^ 0.01 %, *p* = 0.90
			Total R^2^ 7.7 %		Total R^2^ 6.2 %
Subgroup: cartilage measurement site					
Tibia	Tibial cartilage volume	R^2^ 1.7 %, *p* = 0.11	R^2^ 0.8 %, *p* = 0.26	R^2^ 3.34 %, *p* = 0.024	**R** ^**2**^ **3.8 %,** ***p*** **= 0.017**
(*n* = 151)	Meniscal extrusion	**R** ^**2**^ **4.3 %,** ***p*** **= 0.01**	** R** ^**2**^ **3.1 %,** ***p*** **= 0.031**	NA	NA
	Meniscal tears	R^2^ 0.19 %, *p* = 0.60	R^2^ 0.01 %, *p* = 0.91	R^2^ 1.35 %, *p* = 0.15	R^2^ 1.3 %, *p* = 0.16
	Tibial cartilage defects	R^2^ 0.23 %, *p* = 0.56	R^2^ 0.02 %, *p* = 0.85	R^2^ 0.03 %, *p* = 0.83	R^2^ 0.3 %, *p* = 0.52
			Total R^2^ 5.1 %		Total R^2^ 5.1 %
Femur	Femoral cartilage volume	**R** ^**2**^ **3.8 %,** ***p*** **= 0.016**	R^2^ 1.8 %, *p* = 0.095	R^2^ 1.72 %, *p* = 0.11	R^2^ 1.5 %, *p* = 0.13
(*n* = 150)	Meniscal extrusion	**R** ^**2**^ **4.3 %,** ***p*** **= 0.01**	**R** ^**2**^ **3.2 %,** ***p*** **= 0.024**	NA	NA
	Meniscal tears	R^2^ 0.19 %, *p* = 0.6	R^2^ 0.04 %, *p* = 0.80	R^2^ 1.35 %, *p* = 0.15	R^2^ 1.2 %, *p* = 0.19
	Femoral cartilage defects	**R** ^**2**^ **3.0 %,** ***p*** **= 0.035**	R^2^ 2.2 %, *p* = 0.063	R^2^ 2.26 %, *p* = 0.07	R^2^ 2.2 %, *p* = 0.07
			Total R^2^ 9.0 %		Total R^2^ 4.9 %
Subgroup: Radiographic OA of OARSI grade ≥1 (*n* = 85)	Tibial + femoral cartilage volume	**R** ^**2**^ **7.8 %,** ***p*** **= 0.01**	R^2^ 2.1 %, *p* = 0.16	R^2^ 3.0 %, *p* = 0.11	R^2^ 3.3 %, *p* = 0.096
	Meniscal extrusion	** R** ^**2**^ **11.2 %,** ***p*** **= 0.002**	**R** ^**2**^ **6.2 %,** ***p*** **= 0.017**	NA	NA
	Meniscal tears	R^2^ 0.02 %, *p* = 0.91	R^2^ 0.4 %, *p* = 0.53	R^2^ 1.38 %, *p* = 0.28	R^2^ 1.9 %, *p* = 0.21
	Tibial + femoral cartilage defects	R^2^ 4.5 %, *p* = 0.051	R^2^ 2.3 %, *p* = 0.14	R^2^ 0.49 %, *p* = 0.53	R^2^ 1.1 %, *p* = 0.33
			Total R^2^ 16.7 %		Total R^2^ 5.6 %

Results obtained using linear regression

^a^Adjusted for change in cartilage volume, change in meniscal extrusion, change in meniscal tears or change in cartilage defects where applicable

NA indicates not applicable (incidence of meniscal extrusion very low)

Bold text indicates statistically significant result (*p* ≤ 0.05)

R^2^ for *univariable* models is the proportion of variance explained provided (R^2^) for the linear regression

R^2^ for *multivariable* models is the proportion of variance explained for individual components of the multivariable model (semipartial R^2^)

Total R^2^ is the proportion of variance explained for the entire multivariable model

New or worsening meniscal extrusions (ME) occurred in 14 cases (9.2 %) medially and 1 case (0.66 %) laterally. Only the medial data was used in the analysis due to the small number of lateral ME. Of these meniscal extrusions, 9 were none to partial, 3 partial to complete and 2 none to complete extrusion. In unadjusted analyses, change in medial JSW was greater in individuals with new or worsening meniscal extrusions (Table 2, Fig. 3). In multivariable analyses, new or worsening meniscal extrusions over 2.4 years predicted change in JSW over 5 years at the medial tibial (R^2^ 3.1 %, *p* = 0.031) and medial femur (R^2^ 3.2 %, *p* = 0.024) sites, and was strongest in participants with radiographic OA (OARSI grade ≥1) (R^2^ = 6.2 %, *p* = 0.017), but did not reach statistical significance in the whole population at both sites (R^2^ 2.4 %, *p* = 0.053), after adjustment for change in meniscal tears and cartilage defects. When predictive validity of both change in cartilage volume and ME for JSW was considered, they were additive (ie combined R^2^ = 8.3 % in those with radiographic OA).

The addition of cartilage defects or meniscal tears to the model did not reach statistical significance medially or laterally, or within any subgroup.

We repeated analyses using another marker of cartilage assessed in a subset of this cohort: cartilage thickness (Table 3). This data suggests that associations between change in cartilage thickness and change in JSW are of similar or lesser magnitude to change in cartilage volume loss and change in JSW. Similarly, total R^2^ in models assessing change in cartilage thickness (Table 3) is similar to, or less than values for models assessing change in cartilage volume (Table 2). Data on study participants with ROA are not shown as the number of included participants is too small (*n* = 40).

**Table 3:** Association between change in cartilage thickness, and new or worsening meniscal extrusions, meniscal tears, and cartilage defects over 2.4 years; and change in joint space width over 5 years (*n* = 65)

		Medial JSN^a^		Lateral JSN	
		Unadjusted	Adjusted	Unadjusted	Adjusted
Tibial and femoral cartilage combined	Tibial + femoral cartilage thickness	R^2^ 2.4 %, *p* = 0.21	R^2^ 3.6 %, *p* = 0.14	R^2^ 0.2 %, *p* = 0.71	R^2^ 0.0 %, *p* = 0.91
	New or worsening meniscal extrusion	R^2^ 1.5 %, *p* = 0.32	R^2^ 0.4 %, *p* = 0.62	N/A	N/A
	Worsening meniscal tears	R^2^ 0.0 %, *p* = 0.99	R^2^ 0.1 %, *p* = 0.78	R^2^ 1.9 %, *p* = 0.27	R^2^ 1.3 %, *p* = 0.38
	Worsening cartilage defects	R^2^ 2.1 %, *p* = 0.25	R^2^ 1.1 %, *p* = 0.41	R^2^ 1.0 %, *p* = 0.43	R^2^ 0.9 %, *p* = 0.46
			Total R^2^ = 6.2 %		Total R^2^ = 2.3 %
Subgroup: cartilage measurement site					
Tibial cartilage alone	Tibial cartilage thickness	R^2^ 2.5 %, *p* = 0.33	R^2^ 2.5 %, *p* = 0.21	R^2^ 0.7 %, *p* = 0.52	R^2^ 1.7 %, *p* = 0.30
	New or worsening meniscal extrusion	R^2^ 1.5 %, *p* = 0.32	R^2^ 0.8 %, *p* = 0.48	NA	NA
	Worsening meniscal tears	R^2^ 0.0 %, *p* = 0.99	R^2^ 0.2 %, *p* = 0.7	R^2^ 1.9 %, *p* = 0.27	R^2^ 1.3 %, *p* = 0.37
	Worsening cartilage defects	R^2^ 2.1 %, *p* = 0.25	R^2^ 1.3 %, *p* = 0.37	R^2^ 1.0 %, *p* = 0.43	R^2^ 1 %, *p* = 0.43
			Total R^2^ = 5.1 %		Total R^2^ = 3.9 %
Femoral cartilage alone	Femoral cartilage thickness	R^2^ 2.3 %, *p* = 0.22	R^2^ 3.3 %, *p* = 0.15	R^2^ 1.9 %, *p* = 0.27	R^2^ 1.6 %, *p* = 0.31
	New or worsening meniscal extrusion	R^2^ 1.5 %, *p* = 0.32	R^2^ 0.3 %, *p* = 0.65	N/A	N/A
	Worsening meniscal tears	R^2^ 0.0 %, *p* = 0.99	R^2^ 0.1 %, *p* = 0.86	R^2^ 0.4 %, *p* = 0.27	R^2^ 1.6 %, *p* = 0.31
	Worsening cartilage defects	R^2^ 2.1 %, *p* = 0.25	R^2^ 1.0 %, *p* = 0.44	R^2^ 1.0 %, *p* = 0.43	R^2^ 1.1 %, *p* = 0.40
			Total R^2^ = 5.9 %		Total R^2^ = 3.9 %

Results obtained using linear regression

^a^Adjusted for change in cartilage thickness, change in meniscal extrusion, change in meniscal tears or change in cartilage defects where applicable

NA indicates not applicable (incidence of meniscal extrusion very low)

Bold text indicates statistically significant result (*p* ≤ 0.05) (No associations were statistically significant)

R^2^ for *univariable* models is the proportion of variance explained provided (R^2^) for the linear regression

R^2^ for *multivariable* models is the proportion of variance explained for individual components of the multivariable model (semipartial R^2^)

Total R^2^ is the proportion of variance explained for the entire multivariable model

### Sensitivity analyses

X-ray is weight bearing, but MRI is not; therefore we further adjusted for BMI. This increased R^2^ values for change in medial joint space width by small amounts: 3.0 % vs 2.2 % for total cartilage, and 3.0 % vs 2.4 % for meniscal extrusion; and 3.8 % vs 2.1 % for total cartilage, and 6.8 % vs 6.2 % for meniscal extrusion in people with radiographic OA. R^2^ for change in cartilage volume, meniscal extrusion and BMI were 8.3 and 17.1 % for these models.

JSN in one compartment might affect change in another; therefore we assessed change in medial JSN in participants without lateral JSN at baseline (*n* = 126). Total R^2^ values increased by a small amount (7.7 % vs 5.1 %), but R^2^ values for total cartilage volume were similar (R^2^ = 1.8 % vs 2.2 %). R^2^ values for meniscal extrusion increased in magnitude (R^2^ = 4.4, *p* = 0.02 vs R^2^ = 2.4 %, *p* = 0.053), reaching statistical significance.

## Discussion

In this 5 year longitudinal study of a population based cohort, both JSW and cartilage volume decreased significantly over time. Associations between cartilage volume loss and change in JSW at both medial and lateral compartments were weak and did not reach statistical significance at all sites. Associations were consistent in magnitude, explaining 2–13 % of the variance regardless of measurement site or stratified analysis; although not all of these attained statistical significance. The strength of the associations between ME and change in JSW was similar to the associations between change in cartilage volume and change in JSW, and was strongest in those with radiographic OA. Overall, over 80 % of the variation in JSW change amongst individual study participants remains unexplained, possibly due to measurement error.

Whilst we observed an association between change in JSW and cartilage volume loss, the small magnitude of the association suggests that change in JSW over 5 years provides only a very limited reflection of change in cartilage volume over 2.4 years. Unlike radiographs, MRI allows direct visualisation of the cartilage along with other soft tissue structures, and in 3D compared to the 2D for radiographs. MR imaging has been validated in cadaveric studies [35], and has demonstrated a direct link with clinical outcomes such as joint replacement [36].

New or worsening cases of meniscal extrusion were uncommon in our cohort, as previously reported [7]. However, ME contributed a similar or larger amount to change in JSW as cartilage volume change at the medial compartment. Predictive validity of both cartilage volume change and ME for change in JSW was additive, but not entirely independent. In participants with radiographic OA, ME was a stronger predictor than change in cartilage volume for change in JSW at the medial compartment. These changes were observed in relatively small numbers of participants, with only nine participants (5.8 %) having radiographic OA and ME. However, this data is consistent with studies using categorical measures of cartilage and including larger numbers of participants with OA [7].

Adding change in meniscal tears to the multivariable model added minimal additional explanatory power, and did not reach statistical significance at any site or within any subgroup. The proportion of variance explained by cartilage defects were similar to that explained by change in cartilage volume in some subgroups, but the effect did not reach statistical significance in any groups or sites, and the magnitude of the effect was less than meniscal extrusion. Additivivity of factors is limited as increases in R^2^ were modest.

Repeating the analysis using another assessment method for cartilage (average thickness) yielded even weaker associations.

In this study, more than 85 % of change in JSW over time was unexplained by cartilage volume loss, change in meniscal tears, meniscal extrusions or change in cartilage defects. Additionally, the fact that we had to exclude 1/3 of radiographs (>2 mm difference in alignment between films) despite a standardised protocol also suggests the weakness of change in JSW as a measurement over time.

In addition to the factors we measured, the large proportion of unexplained variance could be partially attributed to measurement error due to artefacts in positioning study participants for x-ray. We minimised this through our analysis design, by limiting x-ray data to those whose tibial inter rim distance was ≤2 mm between phase 1 and phase 3 films [37]. Reading of the films themselves is unlikely to add much measurement error, as reproducibility was excellent. Further adjustment for BMI suggests that BMI is not the major source of unexplained variance. Overall, measurement error due to technical issues remains the most likely explanation given that a substantial proportion of subjects actually increased their JSW over 5 years which is unlikely to be physiologic (unless there is greater cartilage loss in the other compartment). The current FDA and EMEA guidelines accept slowing, cessation or reversal of JSN using conventional radiographs as a structural endpoint for pharmaceutical trials of OA therapies, particularly when accompanied by symptom improvement [3]. However, this study raises the question of whether x-ray measures of JSW should remain the gold standard outcome measure in clinical trials, or whether cartilage loss using MRI should be adopted, as is being proposed by others [38, 39].

There have been a number of longitudinal studies comparing cartilage volume loss and JSW [17, 20–23, 32, 40, 41]. Most focus on the medial compartment of the tibiofemoral joint in populations with OA [20, 21, 23, 40]. A cross-sectional association between meniscal extrusion or positioning and JSW has previously been described [4, 7, 22, 42], with one study demonstrating a strong association between medial meniscal subluxation and JSW (r = 0.56) [43], and another showing that meniscal position and change in cartilage score both contributed to JSW over 30 months [7]. However, our study is the first to our knowledge that has investigated associations over 5 years, and has used continuous measures of both cartilage volume and JSW.

There are a number of limitations in this study. The gold standard for X-ray protocols have changed since our study began in 2002, based on evidence suggesting that newer methods may be more sensitive to change in JSW [44]. However, the method we used is a sensitive measure of joint space loss over time [45], and studies have shown no advantage of one flexion x-ray protocol over another [46, 47] although there are no head to head studies [3]. Additionally, the rates of cartilage loss are lower than might be expected in a population of study participants who all had knee OA. However, sensitivity in our community–based sample [18] is comparable [48], or better [17] than SRM’s from other samples. Additionally, we conducted a sensitivity analysis, limiting the data to those with radiographic OA; while the magnitude of the associations increased, the conclusions did not change. Another potential criticism is the method used to evaluate meniscal extrusion. Extrusion was examined on sagittal MRI views, and using the same sequence as cartilage so as to limit the time the participant spent in the MRI scanner. Coronal views may be more sensitive [7], and T2 sequences are better for visualising menisci [4]. Therefore, it can be hypothesised that these analyses may underestimate of the contribution of meniscal extrusion to change in JSW. However, this has never been proven by a head to head study. Additionally, the follow up period for Xrays (5 years) and MRI (2.4 years) was not the same. However, as radiograph–assessed measures of OA progression are less sensitive and require longer periods of observation, this allows additional time for changes that are visible on MR but not on radiographs to become visible, and we do not consider that this affects the conclusions of these analyses. Furthermore, in this study we were only able to examine the sub group of 153 participants in the TASOAC cohort that had full x-ray and MRI data and were adequately aligned. However, because there were no significant differences in baseline factors including demographic factors, anthropometry or imaging abnormalities between the participants included in this study and the whole cohort, this suggests that the validity has not been compromised.

## Conclusions

Change in cartilage volume over 2.4 years only weakly predicted change in JSW over 5 years in participants from a community cohort. ME contributed similarly to cartilage volume change to change in JSW in the medial compartment in all study participants, but a greater amount in those with radiographic OA, while changes in meniscal tears and cartilage defects made minimal contributions to proportion of variance explained. Since MRI examines cartilage directly while radiographs examine it indirectly, these results cast doubt on the validity of using JSW as a proxy measure of loss of cartilage volume. This suggests that it is time to re-evaluate this as the choice of primary outcome measure for clinical trials of disease modifying drugs in OA.
